# *Pet-1* Deficiency Alters the Circadian Clock and Its Temporal Organization of Behavior

**DOI:** 10.1371/journal.pone.0097412

**Published:** 2014-05-15

**Authors:** Christopher M. Ciarleglio, Holly E. S. Resuehr, John C. Axley, Evan S. Deneris, Douglas G. McMahon

**Affiliations:** 1 Silvio O. Conte Center for Neuroscience Research, Vanderbilt University, Nashville, Tennessee, United States of America; 2 Vanderbilt Brain Institute, Vanderbilt University, Nashville, Tennessee, United States of America; 3 Department of Biological Sciences, Vanderbilt University, Nashville, Tennessee, United States of America; 4 Department of Neuroscience, Case Western Reserve University, Cleveland, Ohio, United States of America; Simon Fraser University, Canada

## Abstract

The serotonin and circadian systems are two important interactive regulatory networks in the mammalian brain that regulate behavior and physiology in ways that are known to impact human mental health. Previous work on the interaction between these two systems suggests that serotonin modulates photic input to the central circadian clock (the suprachiasmatic nuclei; SCN) from the retina and serves as a signal for locomotor activity, novelty, and arousal to shift the SCN clock, but effects of disruption of serotonergic signaling from the raphe nuclei on circadian behavior and on SCN function are not fully characterized. In this study, we examined the effects on diurnal and circadian behavior, and on *ex vivo* molecular rhythms of the SCN, of genetic deficiency in *Pet-1*, an ETS transcription factor that is necessary to establish and maintain the serotonergic phenotype of raphe neurons. *Pet-1^−/−^* mice exhibit loss of rhythmic behavioral coherence and an extended daily activity duration, as well as changes in the molecular rhythms expressed by the clock, such that *ex vivo* SCN from *Pet-1^−^* ^/*−*^ mice exhibit period lengthening and sex-dependent changes in rhythmic amplitude. Together, our results indicate that *Pet-1* regulation of raphe neuron serotonin phenotype contributes to the period, precision and light/dark partitioning of locomotor behavioral rhythms by the circadian clock through direct actions on the SCN clock itself, as well as through non-clock effects.

## Introduction

The serotonin (5-hydroxytryptamine, or 5-HT) and circadian systems are two important, interactive regulatory networks in the mammalian brain (for review, see [1]). Both systems exert influence on multiple neural centers to regulate behavior, and originate in specific nuclei in the nervous system. Serotonin, a monoamine neurotransmitter, is synthesized in the brain primarily in neurons of the midbrain and medullary raphe nuclei. The dorsal and median raphe nuclei send projections from the brainstem and midbrain throughout the brain, and interact both directly and indirectly with the central circadian clock, the suprachiasmatic nuclei (SCN) of the hypothalamus, from which they receive reciprocal indirect connections. The serotonergic phenotype of neurons of the raphe requires the expression of *Pet-1*, an ETS proto-oncogene transcription factor that activates the expression of the enzyme tryptophan hydroxylase (TpH2), a key molecule in the 5-HT biosynthetic pathway, as well as other genes that are necessary for serotonergic specification during development [2]. Dysregulation of serotonergic signaling profoundly impacts human mental and physical health, and is a primary contributor to mood and developmental disorders.

Mammalian circadian rhythms are organized by a ∼24 hour biological clock located in the suprachiasmatic nuclei of the hypothalamus, which are composed of some 20,000 tightly packed neurons [3]. Individual SCN neurons can act as self-sustained pacemakers [4], expressing rhythms in spike frequency and gene expression in an autoregulatory timing loop that cycles over the course of a day [5]. The SCN acts to organize the intrinsic rhythms of brain regions and peripheral tissues to regulate behavior and physiology. Like the serotonergic system, circadian dysfunction is associated with a range of sleep, mood, and developmental disorders (for review, see [6], [7]).

In rodents, wheel-running, novelty, and arousal can reset the SCN clock [8], [9], although the clock is set primarily by light [10]. These non-photic behavioral inputs are thought to arise partially from the median raphe nucleus and partially from the intergeniculate leaflet and use serotonin and NPY as neurotransmitters, respectively [11], [12], [13], [14], [15], [16], [17], [18], where the IGL itself receives serotonergic input from the dorsal raphe [19]. SCN neurons themselves express the serotonin re-uptake transporter (SERT) and the serotonin 5-HT_1B_, 5-HT_7_ and 5-HT_2C_ receptors [13], [20], [21], [22], [23], [24], [25], [26] where they mediate serotonin's effects on circadian light responses [15], [27], [28] and circadian rhythms [11], [12], [13], [14], [15], [16], [17]. In the SCN, serotonin modulates glutamatergic light signaling [28] and can phase shift rhythms directly [13], [29] (for review, see [30]). Chemical depletion of serotonin in hamsters, results in maintained rhythms and increases their activity duration in light cycles [31]; likewise, chemical depletion of 5-HT in mice also increases activity duration and alters their ability to entrain to forced treadmill running, even when activity levels are kept constant [32]. Furthermore, behavioral serotonin release, such as that caused by novelty-induced exercise, can inhibit light-induced behavioral phase shifts [33]. Together, these results suggest that serotonin works antagonistically to photic stimulation of the SCN [34], [35], and its depletion in adult rodents alters the clock control of activity in relationship to the prevailing light cycle. The effects on the circadian system of *Pet-1* deficiency and altered serotonin signaling during brain development have not been extensively studied.

In the brain, serotonin signaling is turned on early in mammalian development—E10–12 in mouse [36] or during the first month of gestation in primates [37]. Circadian rhythms, in contrast, appear significantly later during postnatal development. Here, we have used mice with germline knockout of ETS transcription factor *Pet-1* that is necessary for the development of raphe serotonin neurons [38]. *Pet-1* ^−/−^ mice have about an 75% reduction in the number of dorsal and median raphe serotonin neurons that express key serotonin signaling genes, such as Tph2, SERT and 5HT1a. The serotonin projection from the raphe nuclei to the SCN is specifically lost in *Pet-1^−/−^* mice [39], [40].

*Pet-1^−^* ^/*−*^ mice on a mixed genetic background exhibit circadian behavioral abnormalities and abnormal temporal organization [39], [41]. We have examined the effects of this brain specific serotonergic deficiency on diurnal and circadian behavior in a congenic mouse strain (C57Bl/6J), and on *ex vivo* SCN bioluminescence rhythms. Our results indicate that genetic loss of *Pet-1* and serotonergic innervation of the SCN leads to specific changes in diurnal and circadian behavior such as an expansion of activity duration, rhythmic instability, and a treatment-dependent change in free-running period. There are also changes in the period of the isolated SCN *ex vivo*, although these changes are not consistently mirrored by the characteristics of *Pet-1^−/−^* mouse behavior, indicating both SCN and non-SCN effects on circadian control of locomotor behavior by genetic disruption of serotonergic signaling. Sex-dependent effects of the genetic *Pet-1* deficiency on the SCN pacemaker are also evident, suggesting interactions of serotonergic and hormonal regulation of this clock tissue.

## Materials and Methods

### Ethics Statement

All handling of animals was approved by the Vanderbilt University Institutional Animal Care and Use Committee (IACUC) in accordance with National Institutes of Health (NIH) guidelines.

### Animals and Housing

129sv mice containing a Pet-1 null allele [38] were backcrossed twelve generations to C57BI/6J (Jackson) mice to yield a congenic strain for behavioral characterization (males only). These mice were then crossed to a congenic C57Bl/6J Per1::Luciferase [42] reporter mouse line, and both males and females were used for LumiCycle experiments. To avoid maternal-rearing complications from *Pet-1^−^* ^/*−*^ females [43], Pet-1^+/*−*^ males and females were bred to attain *Pet-1* ^+/+^, *Pet-1* ^+/*−*^ and *Pet-1^−^* ^/*−*^ offspring. The breeding *Pet-1* ^+/*−*^ females were not naïve mothers, having had at least one litter prior to the use of subsequent litters in these experiments. For behavioral characterization, mice were bred in a 12∶12 Light:Dark (LD) cycle. Pups were weaned at postnatal day ∼21 and kept together for 1–2 weeks in a litter-filled group cage with food and water ad libitum while they were genotyped as described previously [38]. Animals were then placed individually into litter-filled poly-propylene wheel cages (Coulbourn Instruments, Whitehall, PA) equipped with infrared (IR) motion detector (Spy2 from Visonic, Tel Aviv, Israel) and food and water *ad libitum*. For organotypic slice culture, male and female mice were bred and kept in one of three photoperiods: a short LD 8∶16, an equinox LD 12∶12, or a long LD 16∶8 photoperiod for 50 days. At weaning, mice were housed individually in litter-filled poly-propylene wheel cages with food and water *ad libitum*.

### Behavioral Characterization

**Figure 1:**
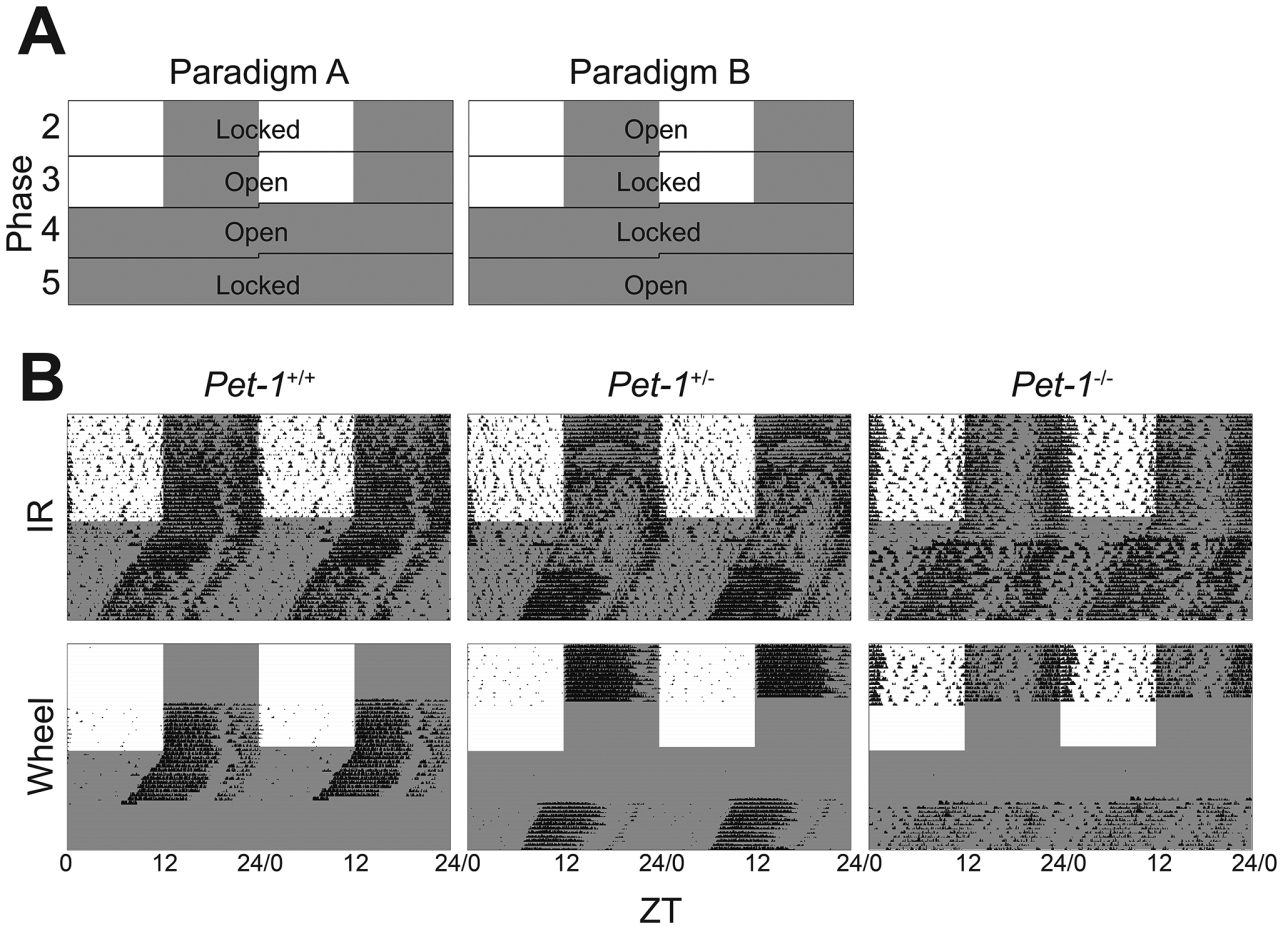
Wheel open/lock circadian behavioral paradigms. **a |** Double-plotted graphics representing two equal light cycle paradigms (12L:12D→DD) with opposite order of wheel access in each phase. **b |** Representative double-plotted actograms showing the monitored activity of mice that are, from *left* to *right*, wildtype, heterozygote, or knockout for *Pet-1*, as monitored by infrared (IR) motion detection (*top*) and wheel (*bottom*). Note that there is no wheel activity during wheel-locked phases. The *Pet-1* ^+/+^ mouse (*left*) represents paradigm A, while the *Pet-1* ^+/−^ and *Pet-1* ^−/−^ mice (*middle* and *right*, respectively) represents paradigm B. Y-axis represents days; X-axis represents time-of-day.

To determine the effect of *Pet-1* deficiency on circadian behavioral properties in conjunction with and independently of wheel activity, we monitored activity in 5-min bins using ClockLab Software (Actimetrics, Evanston, IL) using simultaneous wheel and IR. Specifically, male mice were given 1-2 weeks to acclimate to their cages and entrain to a LD 12∶12 light-cycle (phase 1), after which the mice were split into two groups that were subjected to an identical light paradigm, but with differing wheel-state to control for order-effects. Each animal was subjected to 5 distinct phases in which the mouse had access to a freely turning wheel or in which the wheel was locked (**Figure 1**). The first group (Paradigm A), had the following order of wheel-state: open for about 2 weeks (phase 2), locked for about 3–4 weeks (phase 3: same LD 12∶12 as phases 1–2; phase 4: constant darkness, DD), then finally open in constant darkness for about 2 weeks (phase 5). The second group (Paradigm B) had exactly the opposite order, to test or control for the possibility of order-effects (**Figure 1a**). The final 12 days from each phase were analyzed in ClockLab. We used IR recordings rather than wheel recordings for analysis because it provided activity monitoring during all components of each paradigm.

### Behavioral Analysis

IR behavior was analyzed using ClockLab Analysis software (Actimetrics), and activity was quantified as the number of IR activations occurring during 5-min bins. The free-running period (in DD, over 12 days), rhythmic power, *alpha*, amount of activity during lights-on and lights-off, as well as the total amount of activity per day were determined for each phase using the chi-squared periodogram and activity profile options in the “Batch Analysis” function, and by eye-fit. Phases of activity onset and offset were eye-fit using ClockLab's actogram function. In addition, rhythmic power was determined by using a Chi-squared periodogram that compared amplitude of behavioral rhythms (as in [44]).

### Tissue Preparation for ex vivo Organotypic Slice Cultures

The mice were euthanized by cervical dislocation and rapid decapitation between ZT 9-12. The brains were extracted and blocked in cold sampling media as previously described [45]. 300µm hypothalamic coronal sections containing the suprachiasmatic nuclei (SCN) were cut using a vibroslicer (Campden Instruments Ltd., Lafayette, IN) in Hank's balanced salt solution (HBSS, Gibco, Carlsbad, CA) enriched with 25 U/mL penicillin/streptomycin, NaHCO_3_ (7.5%; Sigma, St. Louis, MO) and 1.0 M HEPES kept at ∼4°C. Slices were then trimmed and transferred to culture membranes (Millicell, Millipore, Billerica, MA) in 35 mm culture dishes containing 1 mL of Dulbecco's modified Eagle's medium – low glucose (DMEM D2902-10x1L; Sigma-Aldrich, St. Louis, MO) supplemented with 25 U/mL penicillin/streptomycin, 1.0 M HEPES, 4.5 g/L glucose, 7.5% NaHCO_3_, N-2 supplement (Invitrogen), and 0.1 mM beetle luciferin (Promega, Madison, WI). Dishes were sealed and placed directly into a LumiCycle (Actimetrics) kept at 35.5°C. Rhythms were analyzed using the LumiCycle data analysis software (Actimetrics) where a baseline subtraction was performed using a fitted polynomial curve to the first six cycles of the raw data with an order value within one cycle of the six cycles to determine the best fit line. The period length was then calculated by determining the maximum and minimum time points (in hours) for *Per1*::LUC expression [42]. Rhythmic power was also examined using the LumiCycle and ClockLab data analysis software (Actimetrics) using a Chi-squared periodogram that compared amplitude to rhythms.

### Statistical Analysis

Means were statistically compared in IBM SPSS 20 (IBM, Armonk, NY) by univariate analyses including repeated measures, with post-hoc tests. For data with non-homogeneous distributions, the Sheirer-Ray-Hare extension of the nonparametric Kruskal-Wallis Test was performed [46]. Post-hoc Fisher's Least Significant Difference tests were performed on parametric data and post-hoc Dunnett's T3 test were performed on non-parametric data. Significance was ascribed at *p*<0.05.

## Results

To determine the influence of genetic disruption of brain serotonergic signaling by *Pet-1* deficiency on circadian behavior and how it may modulate the feedback effects of wheel-running on circadian rhythms, we examined knockout (*Pet-1^−/−^*; N = 16), heterozygous (*Pet-1^+/−^*; N = 14) and wildtype (*Pet-1^+/+^*; N = 9) mice in a series of light/dark cycles combined with locking or unlocking the running wheels available to these individually housed mice (**Figure 1**). We assayed activity both with the running wheel (when unlocked  =  open) and with infrared motion detection. The activity signal readout by infrared motion detectors (IR) persists with both open and locked wheels, and records both on-wheel and off-wheel locomotor activity (**Figure 1**). Visual inspection of the actograms revealed striking alterations in the circadian behavior of *Pet-1 ^−/−^* mice. There were clear differences in the partitioning of day/night activity in LD and in the coherence of free-running rhythms in DD that were quantified with more detailed analysis below.

### *Pet-1^−/−^* mice exhibit expanded locomotor activity duration

**Figure 2:**
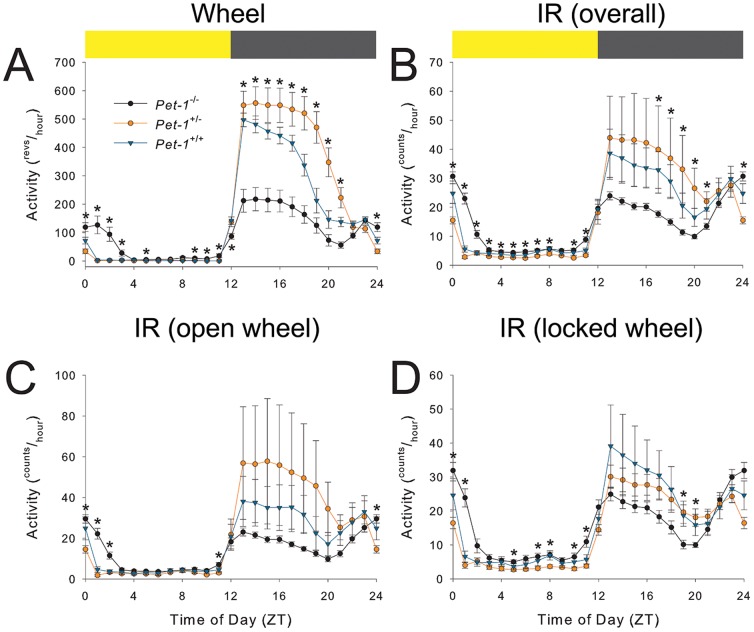
Averaged activity profiles across genotypes as monitored by (a) wheel and (b-d) infrared motion detector (IR) in LD. Each point represents the average across days and across animals within that particular genotype while in a 12L:12D cycle. Blue points represent *Pet-1* ^+/+^ mice; orange points represent *Pet-1* ^+/−^ mice; black points represent *Pet-1* ^−/−^ mice. Colored bar (*top*) represents 24 hour light cycle, where the grey is 12 hours of dark, and the yellow is 12 hours of light. Activity measured in revolutions/hour (a) or counts/hour (b–d). b | Overall IR activity profile of animals regardless of wheel state. These results are further broken-down into IR behavior with a free wheel (c) and with a locked wheel (d). See inset legend (a). Asterisks (*) denote significance at *p*≤0.05. Note the prominent early morning activity in *Pet-1* ^−/−^ mice.

**Figure 3:**
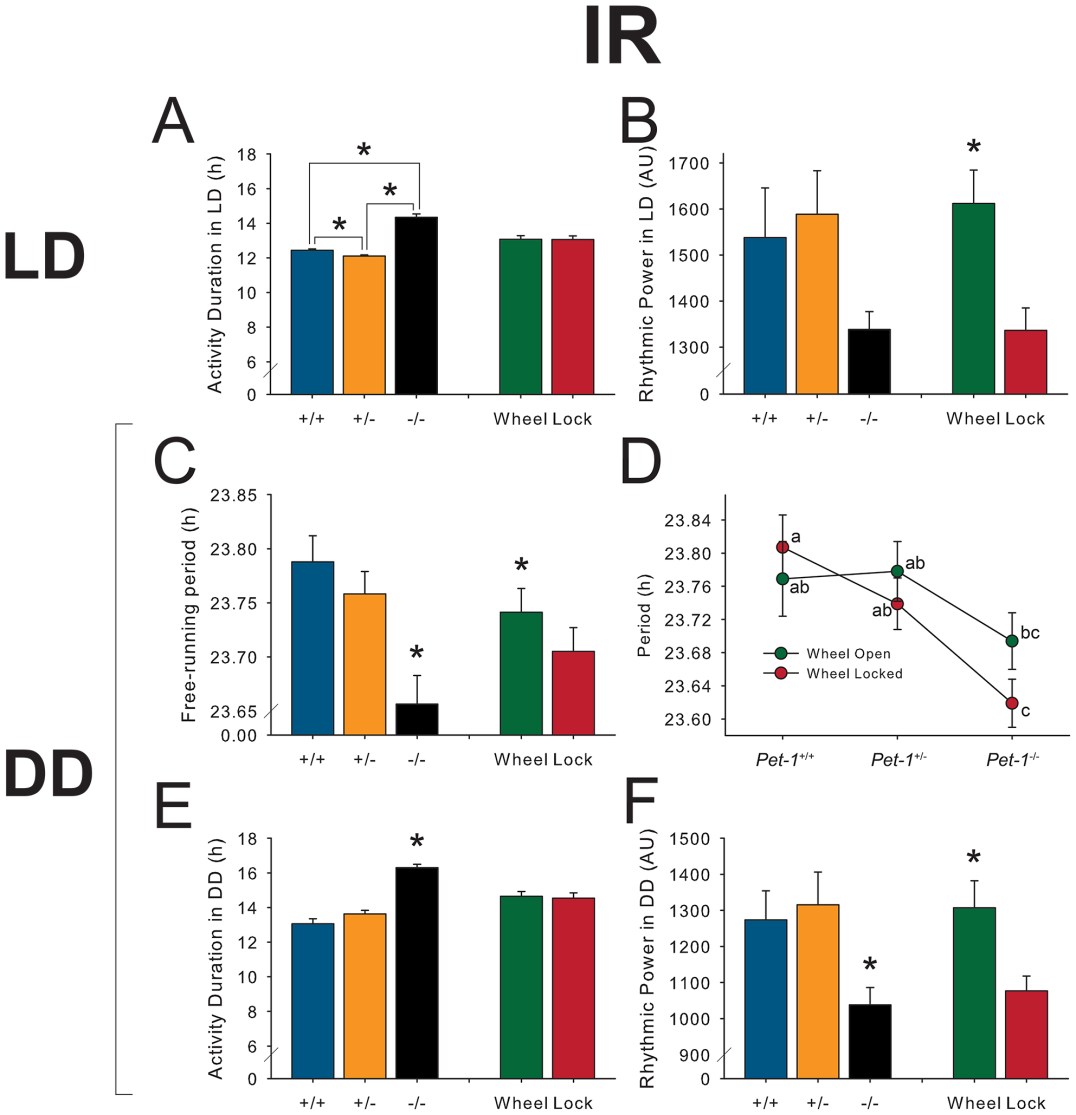
Main effects of *Pet-1* deficiency and running wheel access on circadian behavioral characteristics in 12L:12D (a-b) and constant darkness (c-f) as measured by an infrared motion detector (IR). a | *Pet-1* ^−/−^ mice exhibit a significant increase in length of daily activity duration (*alpha*) in LD. b | Rhythmic power is unaffected by genotype, as measured by χ^2^ periodogram, but exercise leads to a significant increase. c | Free-running period of *Pet-1* ^−/−^ mice is significantly decreased in DD. Furthermore, locking a wheel significantly decreases this period. d | There is a significant interaction between genotype and wheel-access such that as *Pet-1* gene dosage decreases, locking the wheel decreases period to an even greater extent. e | As in LD, *Pet-1* ^−/−^ mice in DD exhibit a greater duration of daily activity. f | *Pet-1* ^−/−^ mice exhibit reduced rhythmic power, and overall, mice allowed to exercise on a wheel exhibit greater rhythmic power. Error bars represent SEM; asterisks (*) denote significance at *p*≤0.05.

**Figure 4:**
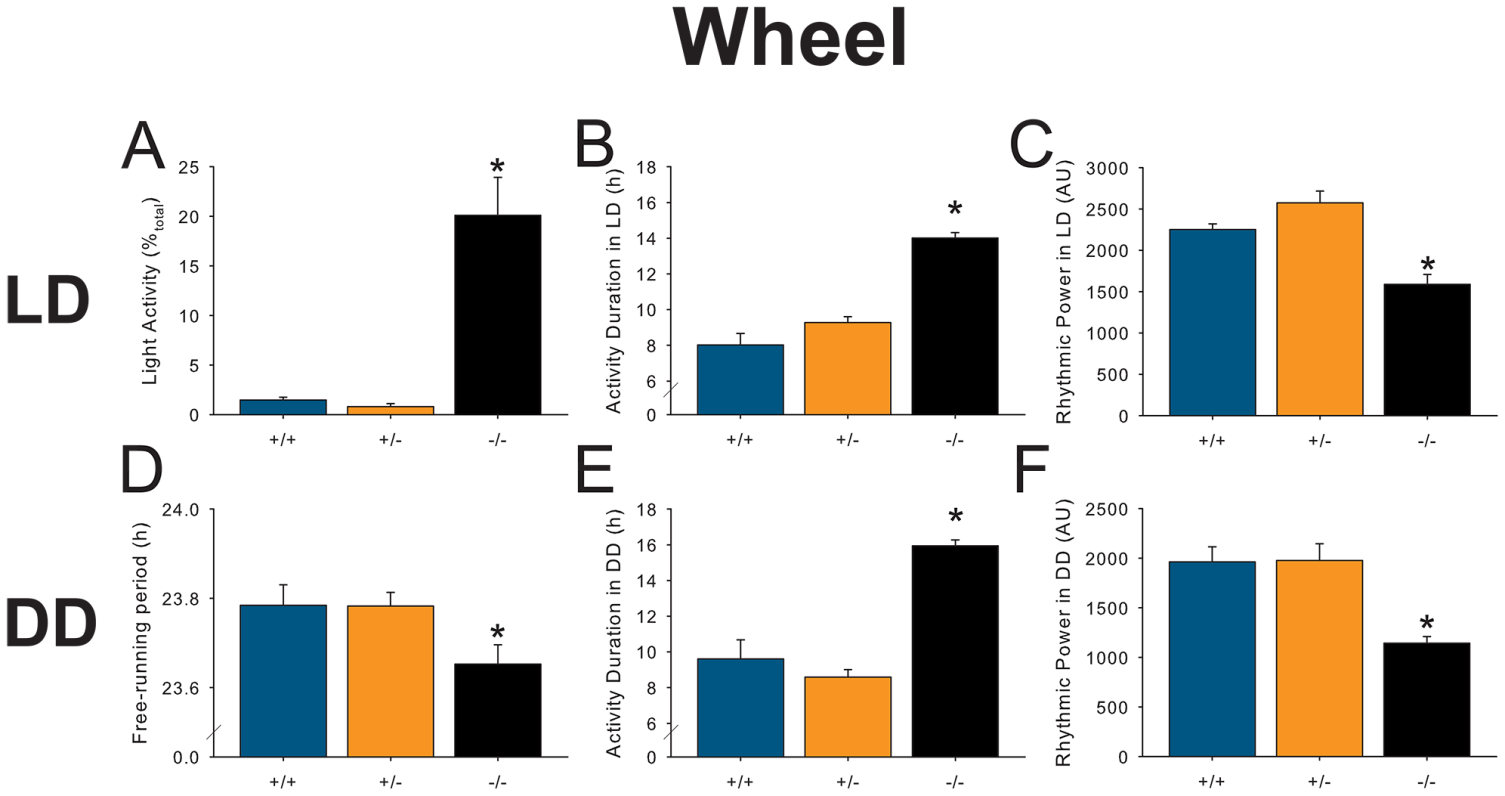
Main effects of genotype on circadian behavioral characteristics in 12L:12D (a–c) and constant darkness (d–f). **a |** The percent of wheel-running activity in the light-phase is significantly increased in *Pet-1* ^−/−^ mice. **b |** *Pet-1* ^−/−^ mice exhibit a significant increase in length of daily wheel-running duration (*alpha*) in LD for *Pet-1* ^−/−^ mice. **c |** Rhythmic power is significantly decreased in *Pet-1* ^−/−^ mice. **d |** Free-running period of *Pet-1* ^−/−^ mice is significantly decreased in DD. As in LD, *Pet-1* ^−/−^ mice in DD exhibit (**e**) a greater duration of daily activity and (**f**) reduced rhythmic power. Error bars represent SEM; asterisks (*) denote significance at ***p*** **≤0.05**.

*Pet-1^−/−^* mice exhibited clear differences in the distribution of activity across the day in LD, with significant decrements in night-time activity and increased day-time activity (**Figure 2**). *Pet-1* ^−/−^ mice exhibited an expanded *alpha*, or activity duration, in both a light-dark cycle (LD) and constant darkness (DD), as measured with both an infrared motion detector (IR; **Figure 3a,e**) and with a wheel (**Figure 4b,e**). Specifically in LD, an earlier activity onset and a later activity offset is an obvious feature of *Pet-1* ^−/−^ mouse locomotor behavior (**Figure 2** **,** **Table 1** **,** and **Figure S1**). Wheel-running did not affect the length of *alpha*, or the timing of activity onsets or offsets in either lighting condition, when compared to the locked wheel condition (**Figure 3a,e** **and** **Table 2**). Pet-1^−/−^ mice exhibited significantly less wheel-running activity overall (**Table 1**), but there was no significant difference in total activity between genotypes as measured by IR (**Table 1**).

**Table 1:** Main effects of genotype (and photoperiod) on circadian behavioral phenotypes.

	Genotype			main-effects		
Characteristic	*Pet-1^+/+^*	*Pet-1^+/−^*	*Pet-1^−/−^*	d.f.	*F* or *Χ^2^*	*p*
*IR Behavioral - LD*						
Onset (ZT)	12.0±0.02	12.0±0.03	11.5±0.09	2	33.856	**<0.001**
Onset_Error_ (h)	0.18±0.01	0.16±0.01	0.30±0.03	2	17.614	**<0.001**
Offset (ZT)	0.4±0.08	0.1±0.06	1.9±0.13	2, 75	2.361	0.101
Offset_Error_ (h)	0.21±0.03	0.22±0.03	0.33±0.02	2, 75	0.802	0.452
Duration (h)	12.4±0.08	12.1±0.07	14.3±0.20	2	57.207	**<0.001**
Power (AU)	1537.4±108.2	1588.1±94.6	1338.2±36.7	2	3.246	0.197
% Light Activity	52.0±3.78	46.7±1.73	53.6±1.08	2	14.543	**0.001**
Average Activity (^counts^/_min_)	1.41±0.25	1.16±0.08	1.16±0.06	2	0.055	0.973
Total Activity (^counts^/_day_)	2044.3±362.5	1698.5±121.5	1675.3±86.6	2	0.027	0.987
Periodic Component Ratio (AU)	0.192±0.03	0.138±0.02	0.480±0.02	2	55.128	**<0.001**
***IR Behavioral - DD***						
Period (h)	23.79±0.02	23.76±0.02	23.66±0.03	2, 75	7.886	**0.001**
Onset_Error_ (h)	0.33±0.03	0.47±0.07	0.47±0.03	2	5.726	*0.057*
Offset_Error_ (h)	0.55±0.03	0.53±0.04	0.60±0.03	2, 75	0.972	0.383
Duration (h)	13.0±0.30	13.6±0.20	16.3±0.20	2, 75	60.131	**<0.001**
Power (AU)	1273.3±81.1	1315.8±90.3	1037.8±48.5	2	8.978	**0.011**
Average Activity (^counts^/_min_)	1.16±0.12	1.11±0.06	1.20±0.07	2	0.706	0.703
Total Activity (^counts^/_day_)	1661.4±178.9	1600.6±83.8	1727.3±97.0	2	0.705	0.703
Periodic Component Ratio (AU)	0.243±0.03	0.268±0.03	0.587±0.03	2,75	86.069	**<0.001**
***Wheel Behavioral - LD***						
Onset (ZT)	12.2±0.03	12.2±0.03	11.7±0.13	2	15.783	**<0.001**
Onset_Error_ (h)	0.09±0.01	0.10±0.01	0.40±0.11	2	7.521	**0.023**
Offset (ZT)	20.2±0.64	21.5±0.31	1.8±0.21	2, 36	73.379	**<0.001**
Offset_Error_ (h)	0.70±0.12	0.85±0.08	0.41±0.07	2, 36	7.915	**0.001**
Duration (h)	8.02±0.64	9.27±0.33	14.01±0.30	2, 36	70.566	**<0.001**
Power (AU)	2251.3±70.7	2575.4±141.2	1589.8±120.1	2, 35	17.832	**<0.001**
% Light Activity	1.47±0.28	0.82±0.29	20.10±3.82	2	25.419	**<0.001**
Average Activity (^revs^/_min_)	13.0±0.88	16.8±1.46	7.5±0.91	2, 35	17.823	**<0.001**
Total Activity (^revs^/_day_)	18790±1273	24151±2108	10865±1312	2, 35	17.823	**<0.001**
Periodic Component Ratio (AU)	0.190±0.01	0.178±0.01	0.397±0.04	2	19.425	**<0.001**
***Wheel Behavioral - DD***						
Period (h)	23.78±0.05	23.78±0.03	23.65±0.04	2, 36	3.771	**0.033**
Onset_Error_ (h)	0.48±0.20	0.36±0.08	0.58±0.07	2, 36	1.227	0.305
Offset_Error_ (h)	0.53±0.07	1.00±0.11	0.58±0.09	2, 36	7.128	**0.002**
Duration (h)	9.61 1.07	8.58±0.43	15.93±0.33	2	27.74	**<0.001**
Power (AU)	1961.7±153.8	1976.3±169.7	1143.0±68.0	2	16.632	**<0.001**
Average Activity (^revs^/_min_)	12.1±0.96	13.0±1.49	7.2±1.02	2, 35	7.337	**0.002**
Total Activity (^revs^/_day_)	7008±3524	952±947	3341±1314	2	1.263	0.532
Periodic Component Ratio (AU)	0.289±0.03	0.252±0.01	0.455±0.03	2	17.922	**<0.001**
***Ex Vivo Per1::Luc Expression***						
Period (h)	24.23±0.07	24.24±0.07	24.50±0.07	2, 155	4.675	**0.011**
Power (AU)	1629.3±76.2	1655.6±74.0	1623.7±79.6	2	0.051	0.951

	Photoperiod			main-effects		
	8L:16D	12L:12D	16L:8D	d.f.	*F*	*p*
***Ex Vivo Per1::Luc Expression***						
Period (h)	24.43±0.07	24.43±0.07	24.11±0.07	2, 155	7.277	**0.001**

Activity monitored by infrared motion detector and wheel in LD and DD, as denoted. *Ex vivo Per1::Luc* expression is below. Significance is ascribed at ***p*** **≤0.05**.

**Table 2:** Main effects of exercise on circadian behavioral phenotypes.

	Exercise		main-effects		
Characteristic	*Wheel Open*	*Wheel Locked*	d.f.	*t* or *Z*	*p*
*IR Behavioral - LD*					
Onset (ZT)	11.82±0.05	11.75±0.08	38	1.338	0.189
Onset_Error_ (h)	0.201±0.019	0.243±0.021	38	−2.605	**0.013**
Offset (ZT)	0.936±0.171	0.844±0.143	38	0.943	0.352
Offset_Error_ (h)	0.283±0.020	0.355±0.021	38	−2.5	**0.017**
Duration (h)	13.07±0.213	13.06±0.207	37	0.047	0.963
Power (AU)	1611.6±72.90	1336.1±48.59	38	2.847	**0.004**
% Light Activity	50.12±1.18	48.32±2.08	38	1.341	0.188
Average Activity (^counts^/_min_)	1.238±0.113	1.196±0.081	38	0.626	0.535
Total Activity (^counts^/_day_)	1803.5±165.1	1734.1±116.4	38	0.703	0.486
Periodic Component Ratio (AU)	0.310±0.03	0.267±0.03	38	2.349	**0.024**
***IR Behavioral - DD***					
Period (h)	23.74±0.022	23.71±0.022	38	2.036	**0.049**
Onset_Error_ (h)	0.420±0.042	0.454±0.042	38	−0.579	0.566
Offset_Error_ (h)	0.512±0.026	0.615±0.030	38	−2.662	**0.011**
Duration (h)	14.64±0.285	14.54±0.309	38	0.535	0.595
Power (AU)	1307.4±74.41	1076.5±41.65	38	−2.805	**0.005**
Average Activity (^counts^/_min_)	1.209±0.071	1.114±0.056	38	1.822	*0.076*
Total Activity (^counts^/_day_)	1736.5±101.8	1596.8±79.3	38	1.849	*0.072*
Periodic Component Ratio (AU)	0.395±0.03	0.383±0.03	37	0.655	0.516

Activity monitored by infrared motion detector in LD and DD. Significance is ascribed at ***p*** **≤0.05**.

### *Pet-1^−/−^* mice exhibit reduced rhythmic behavioral stability in LD

**Figure 5:**
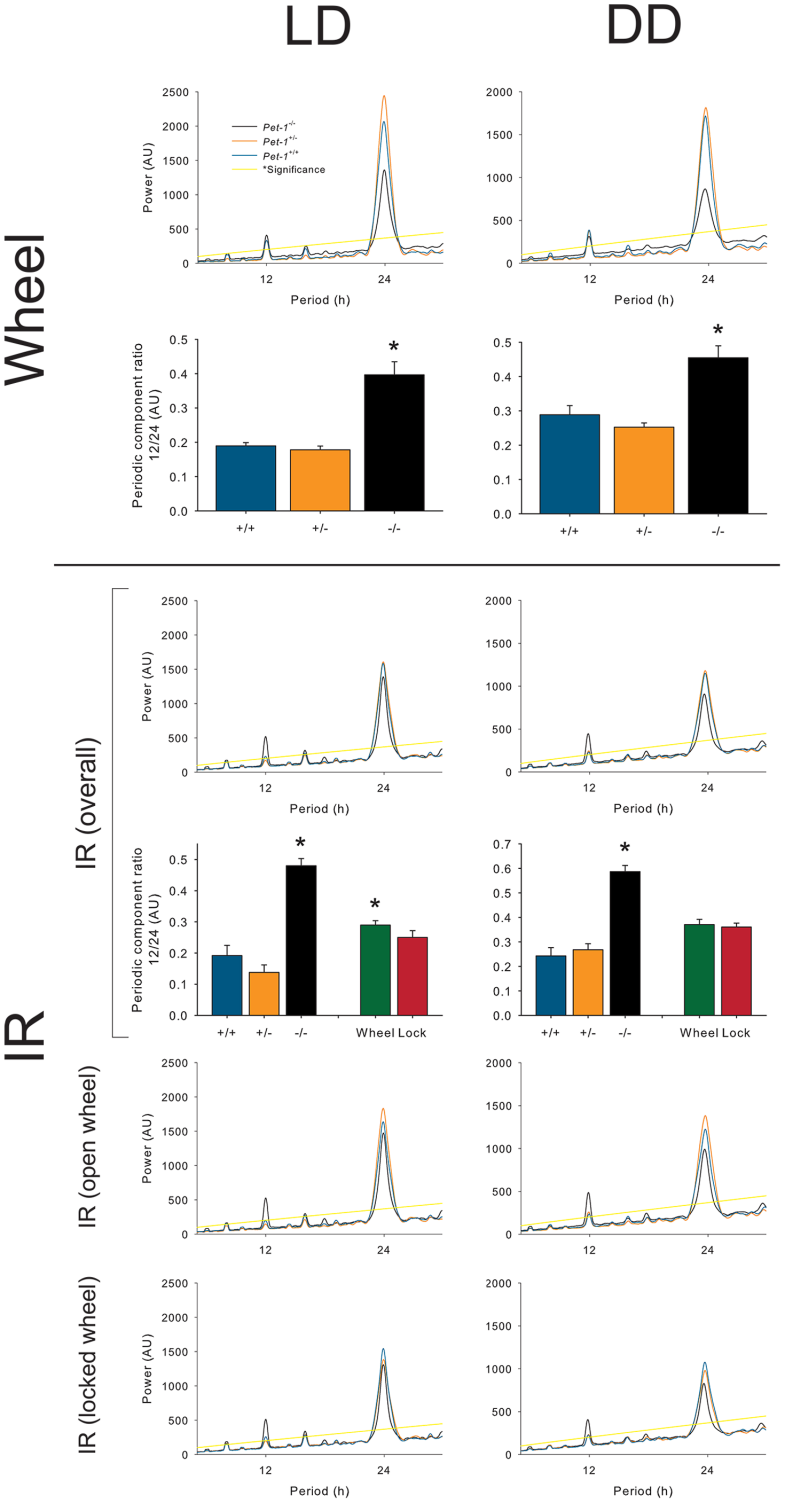
χ^2^ periodogram analyses of genotype-specific and wheel-dependent effects on overall rhythmic amplitude (power) in LD (*top*) and DD (*bottom*). See inset legend (*top-left* panel) for genotype. Purple line represents significance at *p*≤0.05. Periodic component ratios measure the ratio of 12-hour:24-hour components on the wheel. Error bars represent SEM; asterisks (*) denote significance at *p*≤0.05.

In addition to a significantly altered timing of activity onset and offset in relation to the light cycle, *Pet-1* ^−/−^ mice exhibited a change in their day-to-day clock stability in LD. Onset error—the variability of when an animal begins its daily activity—was significantly greater in *Pet-1* ^-/-^ mice in LD, whether measured by IR or wheel-running (**Table 1**). In addition, while all genotypes of mice displayed greater day-to-day activity onset error when their running wheel was locked in LD, the same measure was not different in DD (*p* = 0.057; **Table 1**). *Pet-1* ^−/−^ mice displayed a significant reduction in the coherence of their locomotor rhythms in DD, as measured by rhythmic power (**Figure 3f**), and a similar trend in LD (**Figure 3b**). Wheel-running significantly increased consolidation of rhythms, measured as rhythmic power, in both LD and DD conditions across all genotypes (**Figure 3b,f**). Using a χ^2^ periodogram analyses to measure periodic behavioral components, we found that *Pet-1* ^−*/*−^ mice displayed an increase in the 12-hour component of their activity rhythms leading to significant bimodality of rhythms in a light/dark cycle and in constant darkness (black line, **Figure 5**). This increase in bimodality was present in both the free and locked wheel conditions. When expressed as a ratio of the 12-hour component to the 24-hour component, *Pet-1* ^−*/*−^ mice were significantly different from control mice (**Figure 5**).

### *Pet-1^−/−^* mice exhibit altered behavioral free-running period

In constant darkness, where the SCN's circadian output can be characterized independent of external clock-setting factors (zeitgebers) like light, we found that free-running period was decreased in *Pet-1* ^−/−^ mice by about 0.1 hours (**Figures 3c** **and** **4d**). We did not observe significant shortening of free-running period of any genotype in response to an open wheel (**Figure S2c**), although it has been previously reported that wheel-running shortens free-running period and that effect is dependent on serotonin [11], [14], [47], [48], [49]. We did, however, observe order-effects of wheel exposure (**Figure S2a**) and interactions of wheel-running after-effects on period with genotype that are consistent with the notion that serotonin mediates feedback of wheel-running activity on circadian behavior (**Figure S2, Table S1**).

### *Pet-1^−/−^* mice exhibit altered *ex vivo* SCN free-running period and rhythmic power

**Figure 6:**
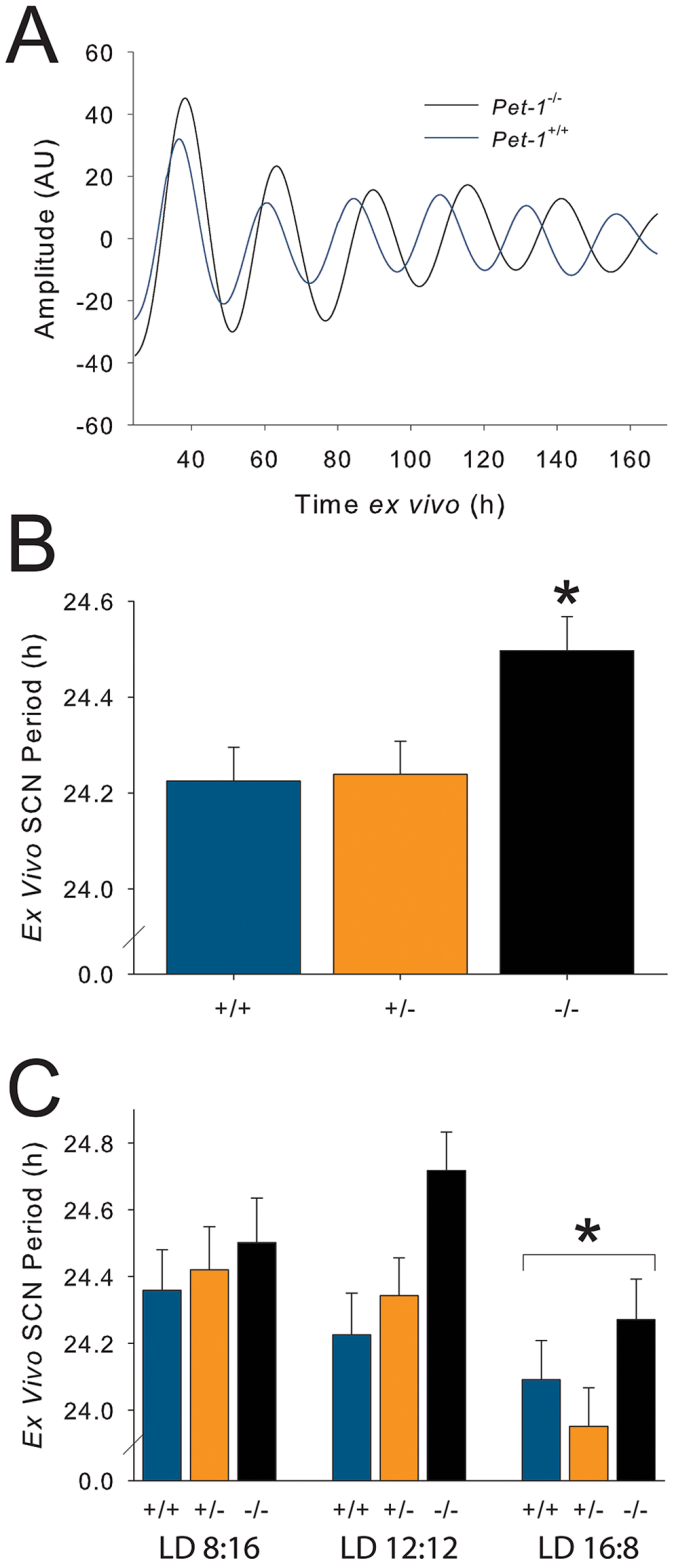
SCN from *Pet-1* ^−/−^ *Per1*::LUC mice exhibit longer period circadian rhythms *ex vivo*. **a | Blue** line is a representative wildtype SCN; **black** line is a representative *Pet-1* ^−/−^ SCN; both taken from a LD 12∶12 cycle. *Ex vivo* SCN period as a function of *Pet-1* genotype (**b**; main effect across photoperiods) or photoperiod (**c**). Asterisks (*) denote significance at ***p*** **≤0.05**.

To examine the properties of the SCN circadian pacemaker in isolation, we crossed the *Pet-1* knockout mouse line with a circadian reporter mouse line in which the rhythmic activation of the *Period1* clock gene promoter is read out as bioluminescence from firefly luciferase (*Per1*::Luc, [42]). SCN tissue explants from wildtype (N = 54), *Pet-1* ^+/−^ (N = 57), and *Pet-1* ^−/−^ (N = 53) genotypes displayed robust circadian rhythms in *Per1*::luciferase gene expression. Analysis revealed that SCN tissue explants from *Pet-1* ^−/−^ mice have significantly longer periods (∼0.25 hours) across six circadian cycles in culture compared to controls (**Figure 6** **,** **Table 1**). We also tested the plasticity of the SCN pacemaker in response to photoperiod, breeding and housing *Pet-1* ^+/+^ *Per1*::Luc, *Pet-1* ^+/−^ *Per1*::Luc, and *Pet-1* ^−/−^ *Per1*::Luc mice in LD 12∶12 (N = 58), 8∶16 (N = 49), and 16∶8 (N = 57) photoperiods and then assaying SCN bioluminescence rhythms *ex vivo*. There was a main effect of the photoperiod such that SCN isolated from *Pet-1* ^−/−^ mice expressed shortened free-running periods of about 0.3 hours following exposure to long, summer-like photoperiods of LD 16∶8 (**Figure 6c**), in agreement with previous results [50].

While only male mice were used in behavioral experiments, as is common practice to avoid the interactive effects of the female estrus cycle on rodent circadian behavioral studies [51], [52], for *ex vivo* experiments, SCN were isolated from the brains of both sexes. As indicated above, sex did not influence the SCN period in culture, however rhythmic power analysis of the *ex vivo* SCN rhythms demonstrated an intriguing phenotype. There was an interaction between sex and genotype such that when a *Pet-1* null allele is present (*Pet-1* ^+/−^ and *Pet-1* ^−/−^), female (N = 28) SCN showed increased rhythmic power while male (N = 24) SCN rhythms exhibited decreased rhythmic power (*p* = 0.029**; Figure S3**).

## Discussion

In this study, we sought to address questions regarding the influence of *Pet-1*, a key gene for central nervous system serotonin signaling on the circadian system. We approached this question by assaying locomotor activity rhythms of mice with genetic knockout of *Pet-1* in LD and DD while simultaneous manipulating their ability to use a running-wheel. We then assayed *ex vivo* cultures of SCN from these mice to assess any differences that a genetic knockdown of serotonin signaling might have on the molecular expression of the rhythm and plasticity in response to photoperiod. Our results indicate that the circadian behaviors of *Pet-1* ^−/−^ mice are significantly altered, but that these changes are not consistently reflected in the isolated clock nucleus, although its properties are also changed.

*Pet-1* ^−*/*−^ mice exhibit significantly expanded locomotor activity over the course of LD 12∶12 light cycles, with increased rhythmic fragmentation and onset error, and increased bimodality in their rhythms. These results are similar to the results obtained with chemical depletion of serotonin in adult hamster [31], in which increased activity duration and fragmentation were also characteristic. While the previous study did not analyze locomotor activity with the same depth, the similarity of results in that study, in which serotonin was only depleted in the adult, and the present study in which serotonin signaling was disrupted during development *and* in the adult, suggests that the effects seen were primarily due to the ongoing depletion of brain serotonin. In addition the similarity suggests that there may be no additional detectable effect of serotonergic signaling disruption on the circadian system during development. Similar results were also reported by Paulus and Mintz [41] in *Pet-1* ^−*/*−^ mice on a mixed genetic background suggesting these aspects of the effects of *Pet-1* deficiency on circadian behavior are robust and consistent. Even with the increase in *alpha* duration, however, there is no increase in total activity over the course of the day in LD or DD (**Table 1**), dispelling concerns that these mice may be hyperactive. Rather, in LD there is a redistribution of activity during the late night and early day. The fact that the most striking effects are seen in rhythms in LD cycles is consistent with the previously described roles and actions of serotonin in modulating SCN light responses. Consistent with this, is a recent report detailing altered acute light-induced clock resetting in *Pet-1* ^−/−^ mice on mixed genetic background [41].

The altered period, duration, and fragmentation of behavioral rhythms suggested the potential for a direct effect on the SCN clock of genetic serotonin deficiency. Interestingly, the rhythmic properties of the SCN itself are indeed modified in *Pet-1* deficiency, perhaps due to loss of serotonin input during development. However, surprisingly, *ex vivo* SCN cultures from *Pet-1* ^−*/*−^ mice did not reveal altered properties consistent with the observed behavioral effects (e.g. shortened period, waveform damping). In fact, the SCN explants showed lengthening of period, the opposite of the behavioral rhythms. This suggests that although *Pet-1* knockout does indeed have genotypic effects on the SCN, those effects are either 1) not preserved in the de-afferented SCN slice, or 2) are preserved, but do not fully account for behavioral effects. There are previous reports of instances in which altered light inputs to the SCN (photoperiod or cycle period) produce opposing effects on the period of circadian behavior and on isolated SCN rhythms [53], [54], [55], [56]. Thus our results are consistent with a loss of *Pet-1* perturbing the regulation of light signaling to the SCN. In addition, our results suggest that the perturbations in circadian behavior are mediated in part by disruption of serotonin signaling to other areas of the brain, since they are not accounted for by the phenotype of the *Pet-1* ^−/−^ SCN itself. We also found that in different photoperiods, the changes in rhythmic period in *ex vivo Pet-1* ^−/−^ SCN cultures agree with previous results on after-effects of proximal photoperiod in wildtype SCN [10], [50], indicating that the SCN pacemaker retains photoperiodic plasticity even in the absence of serotonin input.

A *Pet-1* genotype x sex interaction in setting the rhythmic power of the *ex vivo* SCN suggests an interaction between serotonergic and hormonal regulation of the SCN pacemaker that may differ between males and females. While a number of studies have reported subtle sex differences in behavioral circadian rhythms and clear effects of gonadectomy on circadian organization (for review, see [62], [63]), studies of hormonal effects on SCN physiology are limited [64]. Both estrogenic and androgenic signaling impacts the serotonergic raphe nuclei [52], and thus may modulate the SCN through the serotonergic median raphe projection that is lost in the *Pet-1* ^−/−^ mouse. In particular, estrogen acts to increase the circadian light responses of Raphe neurons [65] and thus may influence SCN properties through this serotonergic circuit. However, androgenic, but not estrogenic signaling can also directly influence SCN neurons, with androgen receptors localized in the SCN core region, which also receives the raphe input [62], [66]. Loss of convergent 5HT/androgenic input to the core region of the SCN in males may account for the sex-dependent effect on SCN rhythms.

It is intriguing that the period change of the *ex vivo Pet-1* ^−/−^ SCN cultures mimics the direction of change seen behaviorally in mixed background *Pet-1* ^−/−^ mice [39], [41], but not that of the C57Bl/6J background. Paulus & Mintz [39], [41] examined the *Pet-1* knockout on a mixed strain of sv129/C57Bl mice that exhibited a significantly longer behavioral period in DD than our results in C57Bl/6J congenic mice. We also behaviorally tested this same mixed strain before backcrossing these mice in our lab, and observed lengthened free-running periods virtually identical to [39], [41] (data not shown), so we are confident that the shortened behavioral free-running period we report here is a phenotype of *Pet-1* ^−/−^ on the C57Bl/6J background strain. Finally, although we did not observe the previously reported shortening of period with wheel activity in wildtype mice, nor its expected loss in *Pet-1* ^−/−^ mice [11], [14], [47], [48], [49], we did observe order-effects of wheel exposure on period indicating that there were interactions between wheel-running and serotonergic tone, but perhaps the relatively short duration wheel exposures we used did not reveal their full extent.

It should be noted that the *Per1*::LUC mouse line we used reports the transcriptional activation of the *Per1* promoter in the circadian transcription-translation feedback loop that forms the core molecular clockworks [42]. Whereas it does not directly report the relative phase of other clock genes, it is subject to transcriptional regulation by them and reports the ongoing period of the molecular clockwork oscillations as a whole. Expression of reporters like the one used here have been shown to correlate highly with neuroelectrical properties and in response to rhythmic perturbations [42], [57], [58], [59], [60], [61].

Here we have demonstrated that a loss of behavioral rhythmic coherence and an extended daily activity duration are robust aspects of the *Pet-1* ^−/−^ circadian phenotype and persist in the face of background strain differences (C57Bl/6J congenic vs. mixed sv129 [39], [41]). These aspects are cardinal features of loss of serotonergic input to the SCN as they are also recapitulated by chemical depletion of serotonin in hamsters [30]. However, specific aspects of the *Pet-1* ^−/−^ phenotype, such as altered free-running period, are dependent on background strain. Finally, we have demonstrated with *ex vivo* SCN explants that loss of serotonergic input to the SCN alters clock period and amplitude (sex-dependent), but that neither the disrupted rhythmic coherence, nor the behavioral period change can be explained by effects of *Pet-1* deficiency directly on the SCN alone because the isolated SCN rhythms do not recapitulate the fragmentation or period change of the *Pet-1* ^−/−^ behavioral phenotype, despite being the same genetic background. Thus, the effects of *Pet-1* ^−/−^ must involve the SCN and other brain regions as well.

Overall, our results suggest that *Pet-1* and its regulation of raphe serotonin phenotype contribute to circadian organization in several distinct ways. Its presence enhances the precision of clock-controlled activity onset and offsets, as well as the coherence of behavioral rhythms. In its absence, activity bouts tend to extend into the light phase of each day, rather than being restricted to the dark phase for nocturnally active mice. In addition, *Pet-1* regulates both behavioral and SCN period, although these effects were not concordant in our study. Disruption of sleep and circadian rhythms is a hallmark of human mood disorders in which deficiency of serotonergic signaling is also implicated. *Pet-1* ^−/−^ mice recapitulate features of circadian disruption typical of mood disorders such as extension of activity into the normal sleep/rest phase (i.e. insomnia) as well as exhibiting increased anxiety-like behaviors [38] and therefore may be a productive experimental system for exploring the relationship between circadian disruption and mood disorders.

## Supporting Information

Figure S1 **Rayleigh plots of activity onset and offset as monitored by IR (** ***top*** **) and wheel-running (** ***bottom*** **), and when the wheel was open (** ***left*** **) and locked (** ***right*** **).** Blue arrowheads represent *Pet-1* ^+/+^ mice; orange arrowheads represent *Pet-1* ^+/−^ mice; black arrowheads represent *Pet-1* ^+/+^ mice; arrows represent the mean phase vector of arrowheads of the same respective color, where length is inversely proportional to the phase variance. Circle represents 24 hours, where the grey is 12 hours of dark, and the yellow is 12 hours of light.
(TIF)

Figure S2 **Mice experience behavioral after-effects in period dependent on genotype and the order in which they gained access to a wheel.** a | Interaction between the order in which mice of all genotypes received access to a wheel and their resultant free-running period during the time the wheel was open or locked. b | Interaction between *Pet-1* genotype and the order in which mice received access to a wheel leads to changes in free-running period. c | While not a statistically significant interaction, this plot illustrates the open/lock components that make up B. Purple icons represent mice that had the wheel open first, then locked; Yellow icons represent mice that had the wheel locked first, then opened. Projection of arrows indicates wheel order-effect. Significance is ascribed at *p*≤0.05.
(TIF)

Figure S3 **Rhythmic power analyses in** ***ex vivo Per1*** **::LUC mouse SCN demonstrates an interaction between** ***Pet-1*** **genotype and sex.**
(TIF)

Table S1 **Descriptive statistics demonstrating interactions between wheel order, wheel access and *Pet-1* genotype, as well as between *Pet-1* genotype and sex.** Significance is ascribed at p ≤ 0.05.
(TIF)
