# Supplementary figures and images for: Pet-1 Deficiency Alters the Circadian Clock and Its Temporal Organization of Behavior
Source: PLoS One. 2014 May 15;9(5):e97412. doi: 10.1371/journal.pone.0097412 (PMC4022518; doi:10.1371/journal.pone.0097412)

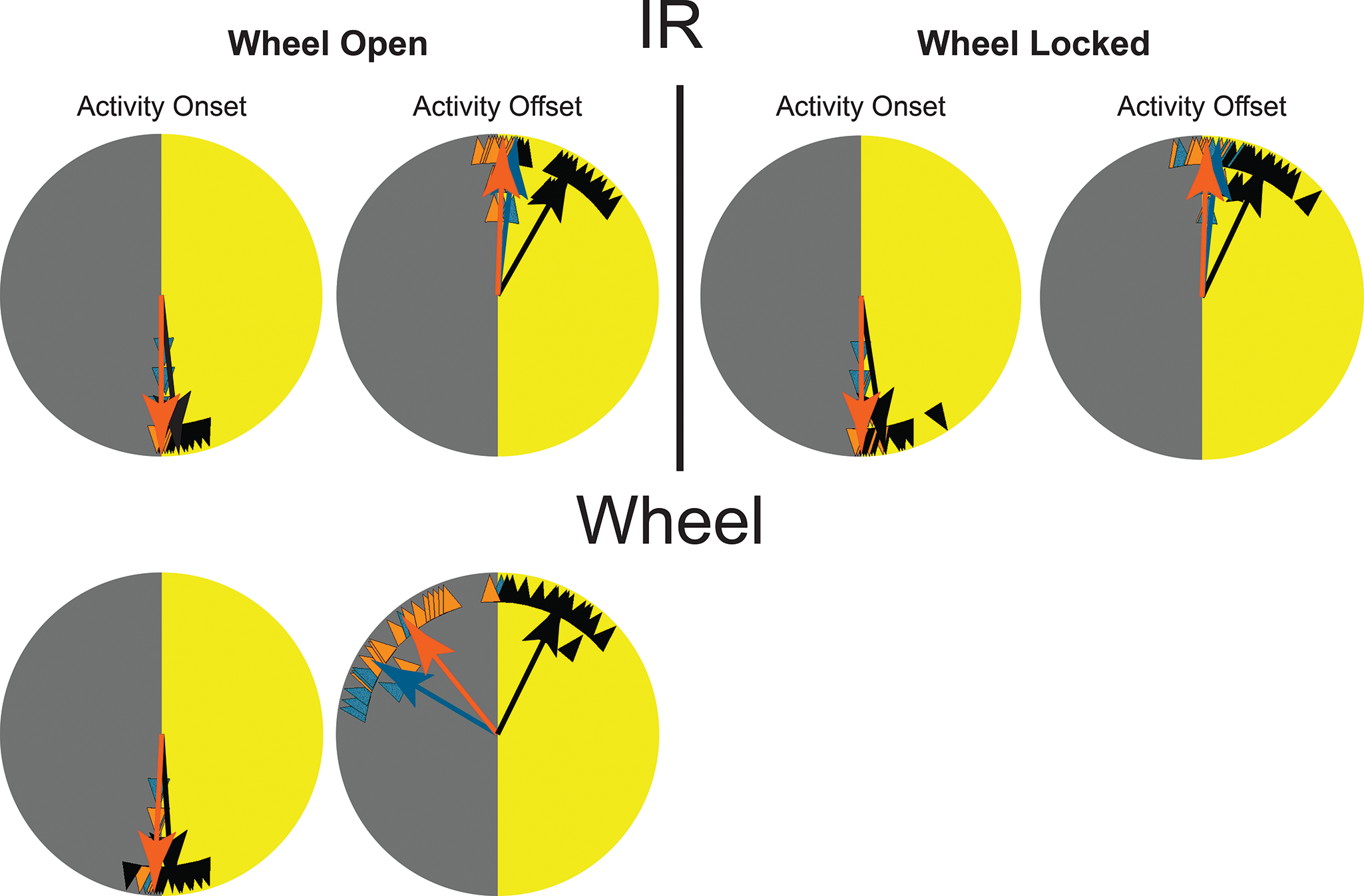

Supplement: Figure S1 — Rayleigh plots of activity onset and offset as monitored by IR ( top ) and wheel-running ( bottom ), and when the wheel was open ( left ) and locked ( right ). Blue arrowheads represent Pet-1 +/+ mice; orange arrowheads represent Pet-1 +/− mice; black arrowheads represent Pet-1 +/+ mice; arrows represent the mean phase vector of arrowheads of the same respective color, where length is inversely proportional to the phase variance. Circle represents 24 hours, where the grey is 12 hours of dark, and the yellow is 12 hours of light. (TIF) [file pone.0097412.s001.tif]

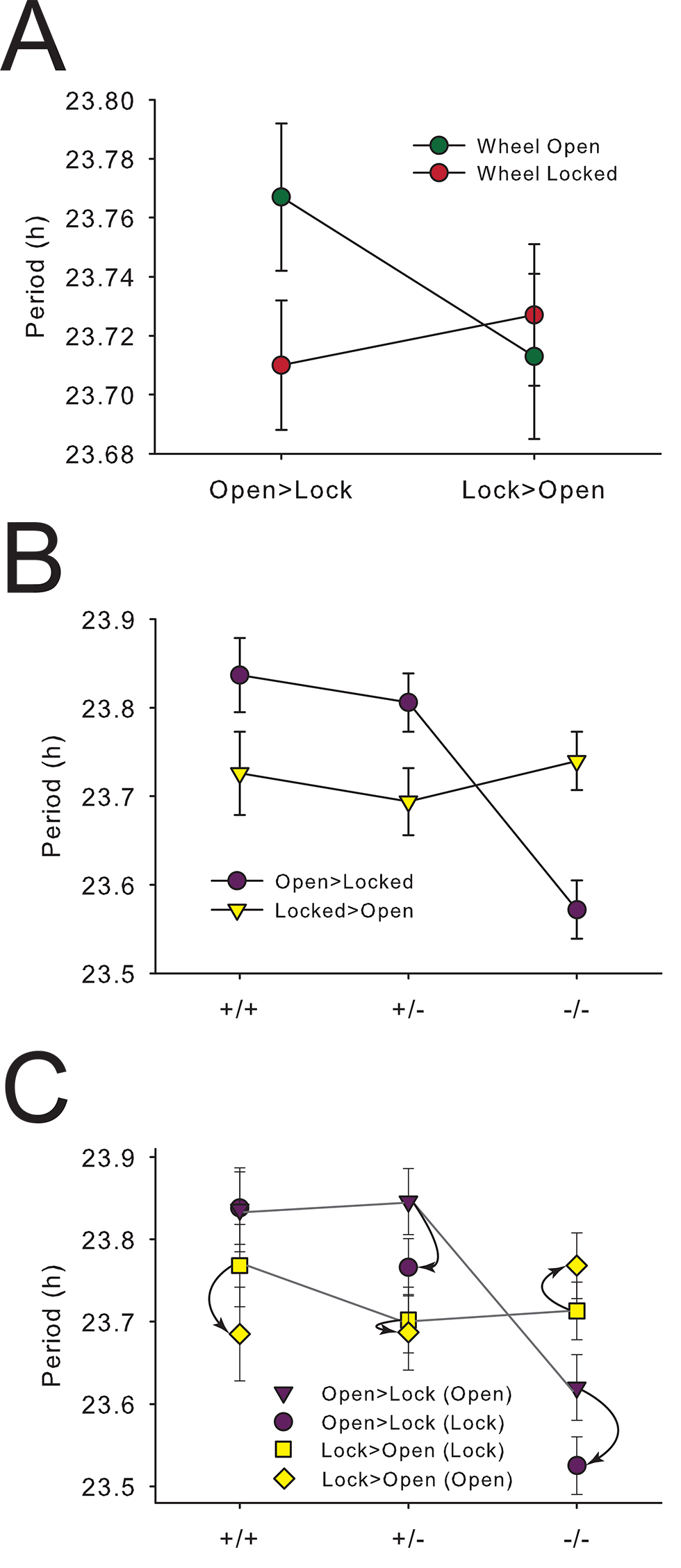

Supplement: Figure S2 — Mice experience behavioral after-effects in period dependent on genotype and the order in which they gained access to a wheel. a | Interaction between the order in which mice of all genotypes received access to a wheel and their resultant free-running period during the time the wheel was open or locked. b | Interaction between Pet-1 genotype and the order in which mice received access to a wheel leads to changes in free-running period. c | While not a statistically significant interaction, this plot illustrates the open/lock components that make up B. Purple icons represent mice that had the wheel open first, then locked; Yellow icons represent mice that had the wheel locked first, then opened. Projection of arrows indicates wheel order-effect. Significance is ascribed at p≤0.05. (TIF) [file pone.0097412.s002.tif]

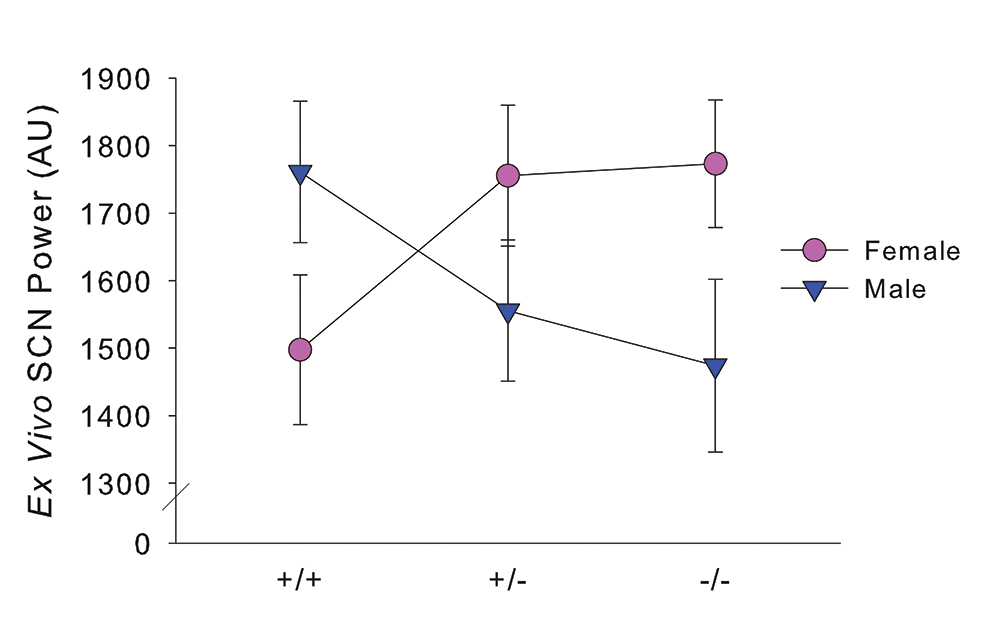

Supplement: Figure S3 — Rhythmic power analyses in ex vivo Per1 ::LUC mouse SCN demonstrates an interaction between Pet-1 genotype and sex. (TIF) [file pone.0097412.s003.tif]

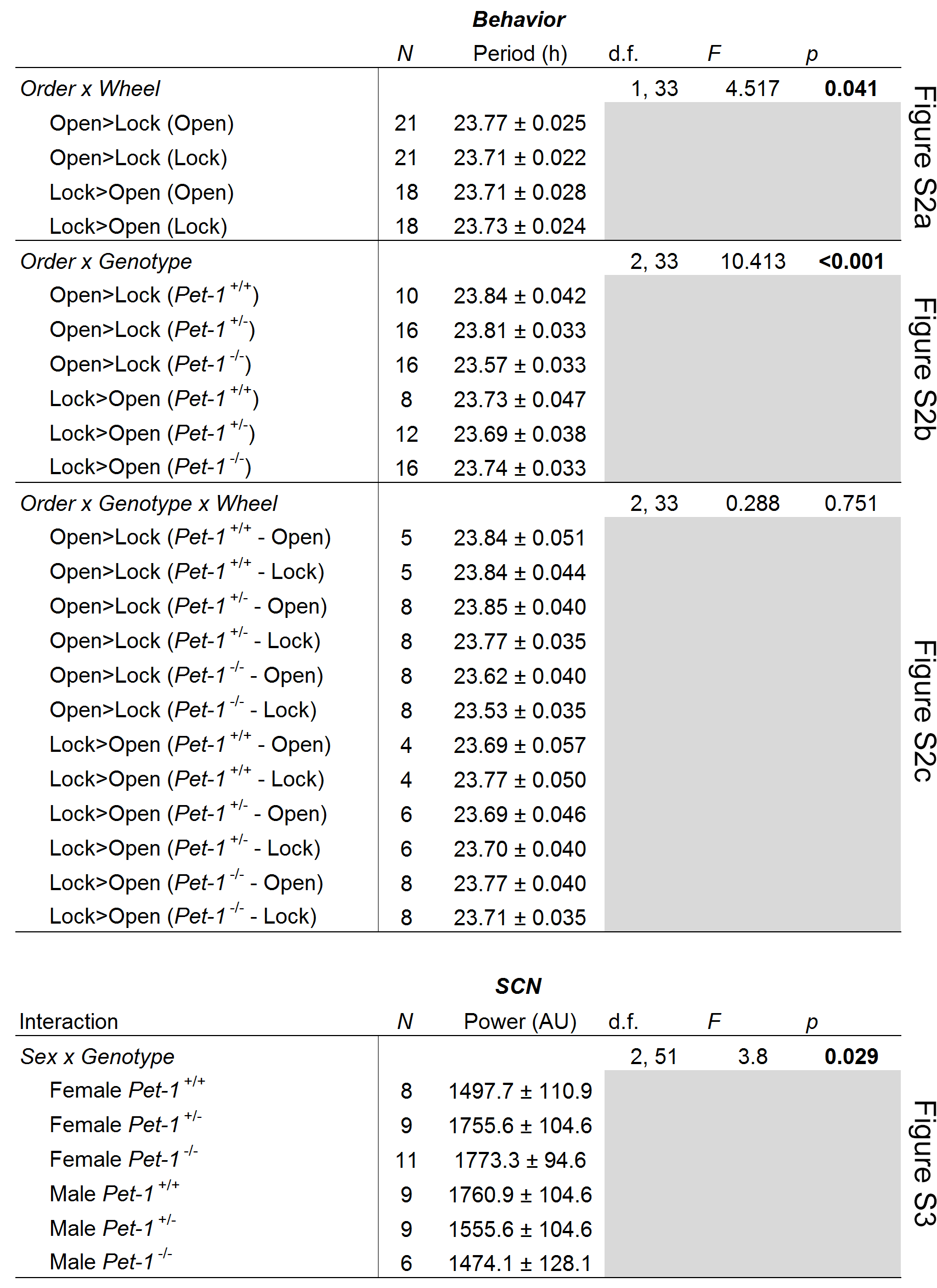

Supplement: Table S1 — Descriptive statistics demonstrating interactions between wheel order, wheel access and Pet-1 genotype, as well as between Pet-1 genotype and sex. Significance is ascribed at p ≤ 0.05. (TIF) [file pone.0097412.s004.tif]
